# Evidence for the Role of Horizontal Transfer in Generating pVT1, a Large Mosaic Conjugative Plasmid from the Clam Pathogen, *Vibrio tapetis*

**DOI:** 10.1371/journal.pone.0016759

**Published:** 2011-02-04

**Authors:** Gaël Erauso, Fatma Lakhal, Adeline Bidault-Toffin, Patrick Le Chevalier, Philippe Bouloc, Christine Paillard, Annick Jacq

**Affiliations:** 1 Laboratoire des Sciences de l'Environnement Marin, UMR 6539, Institut Universitaire Européen de la Mer, Université de Bretagne Occidentale, CNRS, Plouzané, France; 2 Institut de Génétique et Microbiologie, UMR 8621, Université Paris-Sud 11, CNRS, IFR115, Orsay, France; 3 Laboratoire Universitaire de Biodiversité et Ecologie Microbienne, Université de Bretagne Occidentale, Quimper, France; University of Osnabrueck, Germany

## Abstract

The marine bacterium *Vibrio tapetis* is the causative agent of the brown ring disease, which affects the clam *Ruditapes philippinarum* and causes heavy economic losses in North of Europe and in Eastern Asia. Further characterization of *V. tapetis* isolates showed that all the investigated strains harbored at least one large plasmid. We determined the sequence of the 82,266 bp plasmid pVT1 from the CECT4600^T^ reference strain and analyzed its genetic content. pVT1 is a mosaic plasmid closely related to several conjugative plasmids isolated from *Vibrio vulnificus* strains and was shown to be itself conjugative in Vibrios. In addition, it contains DNA regions that have similarity with several other plasmids from marine bacteria (*Vibrio* sp., *Shewanella* sp., *Listonella anguillarum* and *Photobacterium profundum*). pVT1 contains a number of mobile elements, including twelve Insertion Sequences or inactivated IS genes and an RS1 phage element related to the CTXphi phage of *V. cholerae*. The genetic organization of pVT1 underscores an important role of horizontal gene transfer through conjugative plasmid shuffling and transposition events in the acquisition of new genetic resources and in generating the pVT1 modular organization. In addition, pVT1 presents a copy number of 9, relatively high for a conjugative plasmid, and appears to belong to a new type of replicon, which may be specific to Vibrionaceae and Shewanelleacae.

## Introduction

Vibrios are ubiquitous bacterial species in marine ecosystems. They constitute a genetically complex group, associated with a variety of ecological niches, as free-living organisms or in association with various hosts, the interactions ranging from symbiosis to pathogenicity. The number of new species in the *Vibrio* genus keeps increasing with more than 124 species described to date [1]. There has been so far no good correlation between taxonomic identification and pathogenesis, suggesting that virulence mechanisms and host specificity factors might have evolved through horizontal gene transfer rather than through host-bacterium co-speciation [2], [3]. Several Vibrios such as *V. cholerae*, *V. vulnificus* and *V. parahaemolyticus* are human pathogens. However, the majority of *Vibrio* species are symbionts, commensals or pathogens of fish and shellfish and among bacterial diseases, vibrioses are arguably the most important infections in mollusks (Cf. review by Paillard [4]). For instance, in the case of the Japanese clam *Ruditapes phillipinarum*, the etiology of a new disease, the “Brown Ring Disease” was demonstrated by Paillard et Maes [5] to be a new *Vibrio* species, named *Vibrio tapetis*, with the type strain (CECT4600^T^) isolated in Landéda (France) from cultured clams [6]. Shortly after the intentional introduction of *R. phillipinarum* on the Brittany coast of France, *V. tapetis* was responsible for several episodes of winter mass mortalities, causing important economic losses [5]. Since 1990, forty *V. tapetis* strains have been isolated in Europe and in Korea from various bivalve species displaying Brown Ring Disease symptoms and from moribund specimens of two fish species, wrasse and halibut [7], [8], [9], [10], [11]. Analysis of 20 isolates from various geographical and host origins showed that *V. tapetis* strains constitute a homogenous group sharing similar biochemical, antigenic and genetic properties (Christine Paillard, unpublished results).

Plasmids are frequently important components of virulence in bacteria. They can either carry genes encoding toxins, pathogenic traits such as type III (TTSS), type IV (T4SS) secretion systems, regulators of expression of virulence genes, and genes important for the adaptation of the bacterium to its host (such as those involved in iron metabolism) or for antibiotic resistance. A classical and well studied example is the virulence plasmid of *Yersinia pestis*, pYV, which encodes a TTSS and several anti-host effector proteins [12]. Plasmid pWR100 of *Shigella flexneri* [13] also encodes a TTSS system required for entry into epithelial cells and apoptosis induction in macrophages as well as an iron-sequestering system [14]. Other examples of virulence plasmids include the plasmids of non-typhoid *Salmonella* strains [15]. In *Vibrio anguillarum* (now renamed *Listonella anguillarum*), a 65 kb plasmid called pJM1, was found to be essential for virulence due to an encoded iron-sequestering system required for adaptation to the fish host [16], [17], [18], [19]. A more recent example is provided by the two highly related plasmids pR99 and pC4602-2 from two strains of *V. vulnificus* serovar E, CECT4999 and CECT4602 respectively, which were shown to be essential for virulence to eels [20], [21]. In the shrimp pathogen *Vibrio nigripulchritudo*, two plasmids are important for full virulence [22], [23]. Finally, plasmids can confer important adaptive traits to pathogens, for instance when they carry antimicrobial resistance genes. For instance, in an *Aliivibrio salmonicida* strain isolated from a fish farm, a 170-MDa R plasmid was found to carry a tetracycline resistance gene [24].

Other smaller *Vibrio* plasmids such as pMP1 and pVC encode proteins with either homology to phage proteins or without homologs in the databases [25], [26]. Whereas these two plasmids have features characteristic of theta-type replicons, other recently described *Vibrionaceae* plasmids belong to a newly described ColE1-like plasmid group [27], [28]

In the *V. tapetis* reference strain CECT4600^T^, Castro *et al* described a large plasmid, estimated to be around 75 kb [29]. In a later study, Le Chevalier *et al.* described in all the analyzed strains of *V. tapetis* the presence of at least one large plasmid of about 80 kb [30]. In addition, these authors suggested that those strains could contain up to four large plasmids ranging from 60 to 100 kb. Considering the potential importance of such plasmid(s) in *V. tapetis* physiology, we decided to determine the complete sequence of *V. tapetis* CECT4600^T^ plasmid(s). Our study allowed us to identify in the reference strain one plasmid of 82, 266 bp, named pVT1, the analysis of which is the objective of this report.

## Methods

### *V. tapetis* growth, DNA isolation and analysis

Type strain CECT4600^T^, containing the pVT1 plasmid, was grown overnight at 18°C with aeration in Saline Luria-Bertani (LBS) broth (10 g Bactopeptone, 5 g Yeast extract, 20 g NaCl per liter). Plasmid DNA was prepared using the Qiagen large-construct kit (Venlo, Netherlands). Restriction analysis was performed using standard protocols [31]. Electrophoresis in a 0.4% agarose gel was carried out at 40 volts overnight and the gel was stained by ethidium bromide after electrophoresis. For Pulse Field Gel Electrophoresis (PFGE) analysis, 40 µl of low melting point agarose (LMP, Seakem, Rockland, ME, USA) at 1.5% was added to 20 µl of restriction reactions. After gelling, the plugs were placed in the wells of a 1% LMP agarose gel. Migration was for 15 h at 170 mA with 1 pulse/sec in 0.5 X TBE, as described previously [32].

### Cloning of pVT1, shotgun cloning and DNA sequencing

To generate pBac17, pVT1 DNA was digested by NotI and religated between the two NotI sites of pBeloBac11 [33]. Both pBac17 and pVT1 were used as DNA sources for sequence determination. Plasmid DNA was further purified by ultracentrifugation in a cesium chloride gradient in the presence of ethidium bromide (1 mg/ml) [31]. DNA was sheared with compressed air by using a nebulizer (Invitrogen, San Diego, CA, USA), generating fragments of about 1 to 2 kb. After blunt-end repair and dephosphorylation, using the End-Repair kit from Epicentre (Madison, WI, USA), the fragments were religated into the SmaI site of pUC28 [34] and the recombinant clones were introduced into *Escherichia coli* X-Gold (Stratagene, La Jolla, CA, USA). Subsequent blue/white screening was used to detect transformants carrying an insert. Plasmid DNA from positive clones was isolated with a miniprep kit (Qiagen). Library clones were sequenced using facilities at the sequencing platform of the Genopole Ouest (Roscoff, France), using M13 forward and M13 reverse primers. Sequences were trimmed and assembled using the Seqman II program (Lasergene package, DNA Star Inc). Using specific primers to sequence PCR amplicons obtained with native pVT1 as a template, the remaining gaps were filled and the coverage of specific regions was improved, whenever required. Assembly using approximately 2,000 sequencing reads led to a single contig including 1,222 reads, with an average coverage of 9.5 fold, with both strands completely sequenced and a minimal three-fold coverage.

### Bioinformatics analysis

DNA sequences were analyzed using Seqbuilder (Lasergene package) and Vector NTI software (version 10.3). Potential coding sequences were further predicted using GeneMark.hmm-P, using an heuristic model option (http://exon.gatech.edu/GeneMark/heuristic_hmm2.cgi) [35] and AMIGene (www.genoscope.cns.fr/agc/tools/amigene/). Predicted ORFs of more than 50 amino acids were retained. In addition, an ORF not predicted by these programs but encoding a 130 amino acid polypeptide (pVT1_29) was also included in the analysis. Homology searches were performed with a range of Blast tools at the NCBI server (http://www.ncbi.nlm.nih.gov/blast). Genes having similarities with genes encoding transposases were further analyzed by Blast at the IS finder web site (http://www-is.biotoul.fr/). Identification of potential signal peptides was done with SignalP (http://www.cbs.dtu.dk/services/SignalP). Transmembrane domains were predicted by the program TMHMM (http://www.cbs.dtu.dk/services/TMHMM/). Putative lipoproteins and their localization were predicted using LipoP 1.0 (http://www.cbs.dtu.dk/services/LipoP/) [36]. Potential localization of peptides was predicted using Psort (http://psort.hgc.jp/form.html). Comparative genomic analyses of pVT1 with related plasmids were performed using tools proposed by the annotation platform MicroScope at the Genoscope (Evry, France) [37]. The phylogeny of plasmid putative Rep proteins was established using the web server http://www.phylogeny.fr/ in default mode. Phylogeny.fr runs and connects various bioinformatics programs to reconstruct a robust phylogenetic tree from a set of sequences: sequences are aligned using Muscle, the alignment is curated using G-blocks, the phylogeny established using PhyML-aLRT and the tree drawn using TreeDyn [38].

### Plasmid copy number determination

Copy number was determined by measuring the ratio of plasmid gene *traC* (forward primer: ATCCACGCTTCCAGCAA, reverse primer CCCACAACAAACTGACCCAAA) and *traG* (forward primer GGGCACACCTACACGCAAA, reverse primer TGGCTGCTGTCATAGGTCATG) to the chromosomal *gyrB* gene (forward primer CGTCAAGGCTCTACGCATCA, reverse primer GCACCAAGTGGCGCATCT) by real time PCR. Total DNA was prepared from 45 ml of a culture of *V. tapetis* grown to late exponential phase. Cells were resuspended in 800 µl of TNE (100 mM Tris-HCl, 1 mM EDTA, 0.1 M NaCl, pH 8), 20 µl of RNase at 5 mg/ml was added before treatment by lysozyme at 1 mg/ml, prior to lysis in the presence of 1% SDS and 1% Sarkosyl. After centrifugation at 6000× g at 4°C for 15 min, the supernatant was treated by proteinase K at 55°C for 1 hour. This treatment was followed by phenol/chloroform extraction and the DNA was finally precipitated with isopropanol [31]. Plasmid copy number was determined by quantitative Real Time PCR, using three different concentrations of total genomic DNA as template, with three independent experiments for each concentration, using a 7300 Real-Time PCR System (Applied Biosystems, Foster City. CA, USA), as described by Providenti *et al* [39]: ΔCt was determined using the y-axis intercept method and the copy number calculated using F^ΔCt^, where F = 2 if the efficiency is 100%.

### Introduction of a *cat* gene in pVT1

To facilitate conjugation experiments involving pVT1, and provide a mean of selection, the pSW23T suicide vector, carrying a CmR resistance marker [40] was introduced into ORF pVT1_36, which encodes a transposase of the IS4 family (Table S1), as previously described [41], generating strain VT14 (CECT4600 *pVT1_36::pSW23T*).

### Conjugation experiments

Conjugation using VT14 as the donor strain and *Vibrio harveyi* LMG 7890 [42] or *E. coli* DH5α-T1 (F-φ80 *lacZ* ΔM15 Δ(*lacZYA-argF*)U169 *recA1 endA1 tonA hsdR17* (r_K−_, m_K+_) *phoA supE44* λ -*thi-1 gyrA96 relA,* Invitrogen) as the receiver strain was performed by filter mating [43]: 10^7^ exponentially growing cells of donor and receiver strains (or receiver alone and donor alone as negative controls) were incubated on a filter overnight on an LBS plate at 18°C, in triplicate. Cells were eluted from the filters in 1 ml LBS and 100 µl were spread on LBS +2 µg/ml chloramphenicol (*V. harveyi*) or LB+20 µg/ml chloramphenicol (*E. coli*) and incubated at 37°C. This temperature was effective to counter-select *V. tapetis* since this species does not grow above 25°C. Putative exconjugants were purified twice on the same medium, before genomic and plasmid DNA extraction for further analysis.

## Results and Discussion

### Nucleotide sequence of pVT1

**Figure 1:**
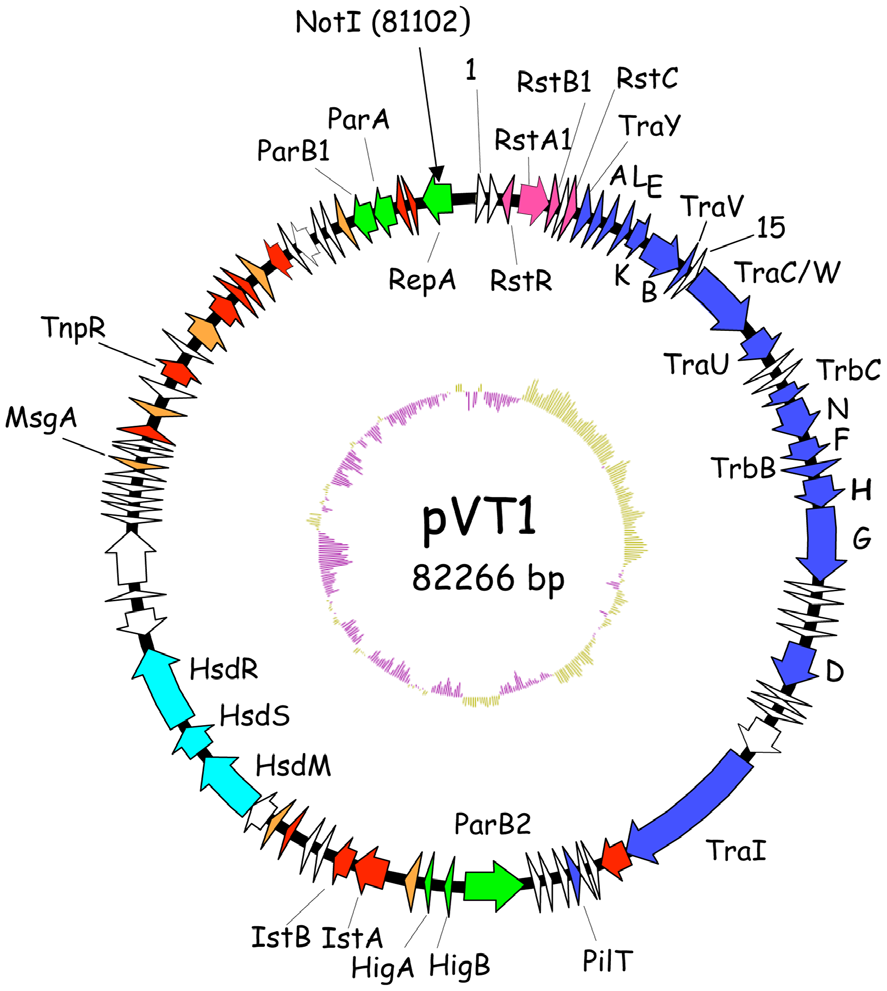
Genetic organisation of pVT1. This diagram shows the circular genome of pVT1. ORFs are shown as arrows. ORFs involved in conjugation are filled in blue, ORFs from the defective phage RS1 in pink, ORFs involved in plasmid maintenance and replication are in green. The three ORFs corresponding to the Type I modification/restriction system are indicated in light blue. ORFs encoding transposases or remnants of transposases are filled in red. ORFs with some indication of functions (with the exception of transposases) are filled in orange. Other ORFs are not filled. The position of the unique NotI site is indicated. The deviation of the G+C content to the mean, calculated with a window of 500nt, is indicated in the inside track (in yellow, G+C> mean, in purple G+C content < mean.

Digestion of pVT1 by NotI resulted in a single band around 85 kb in size, as determined after pulse-field gel electrophoresis (data not shown). pVT1 digested by NotI was religated between the two NotI sites of pBeloBac11 [33], giving pBac17. pBac17 DNA as well as cesium chloride preparation of pVT1 DNA from *V. tapetis* were used for sequence determination. pVT1 is 82,266 bp in size and contains 88 predicted ORFs of more than 50 amino acids (Table S1 and Fig. 1). ORFs smaller than 50 amino acids either presented no similarity to known proteins or corresponded to pseudogenes (see also below). The average G+C content is 45.4%, comparable with the 43.2% reported for the *V. tapetis* reference strain genome [6]. However, the G+C content distribution is highly heterogeneous along the plasmid DNA, varying from 38% to 51% (see below and Fig. 1). This suggests acquisitions in the plasmid of discrete elements of distinct origins, via horizontal transfer. Eight ORFs correspond to proteins predicted to be involved in plasmid maintenance functions. There was no indication of function for 38 ORFs, of which 18 have no homologs in the database.

### pVT1 is a mosaic plasmid containing numerous mobile elements

**Figure 2:**
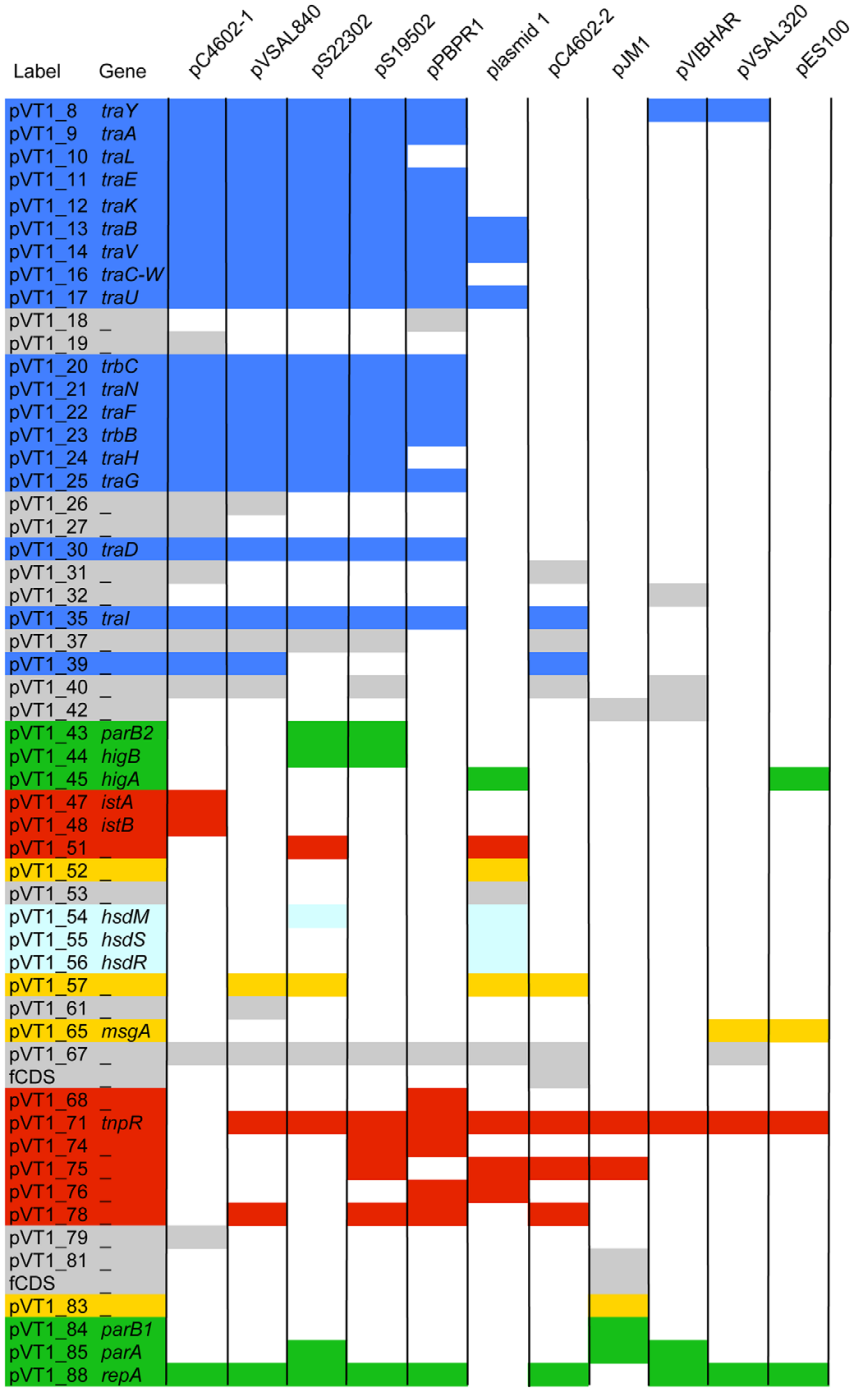
Relationships between pVT1 and a subset of plasmids from marine bacteria. Reciprocal BlastP was used for each pVT1 ORF to determine the presence of a homolog on a given plasmid with an identity threshold >25%. pC4602-1, pC4602-2: plasmid from *V. vulnificus* CECT 4602; pVSal840, pVSal320: plasmids of *A. salmonicida* LF118; pS19502: plasmid from *S. baltica* OS195; pS22302: plasmid from *S. baltica* OS223; plasmid 1: *Shewanella* sp. ANA.3 plasmid 1; pBPR1: *P. profundum* SS9 plasmid 1; pJM1: plasmid from *L. anguillarum*; pVIBHAR, plasmid form *V. harveyi* ATCC119; pES100: plasmid from *V. fisheri* ES114. The colour code is identical to Fig. 1, except for genes with unknown function filled in grey. No filling indicates the absence of a homolog on a given plasmid. The result for pYJ016 was ommited because it was very similar to the comparison with pC4602-1.

**Figure 3:**
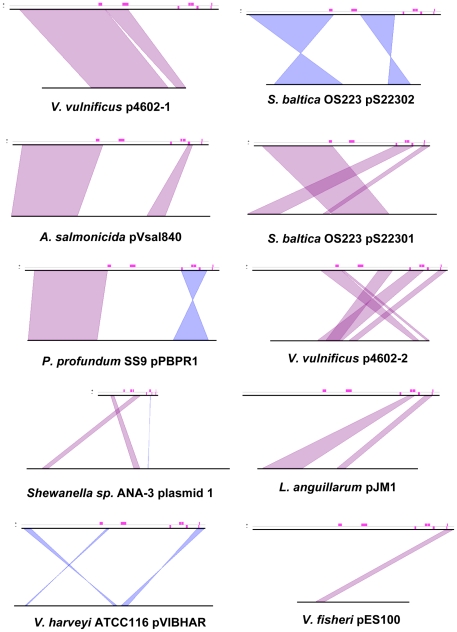
Conservation of synteny between pVT1 and related plasmids from marine bacteria. pVT1 is represented on the top line, the compared plasmid on the bottom line. Pink rectangles above the top line indicate positions of IS elements or remnants of IS in pVT1. Conserved blocks of synteny between the two compared plasmids are indicated in purple, if the genes are present on the same strand or in blue if the genes are expressed on the opposite strand. See also the text and legend to Figure 2 for details on plasmids.

**Figure 4:**
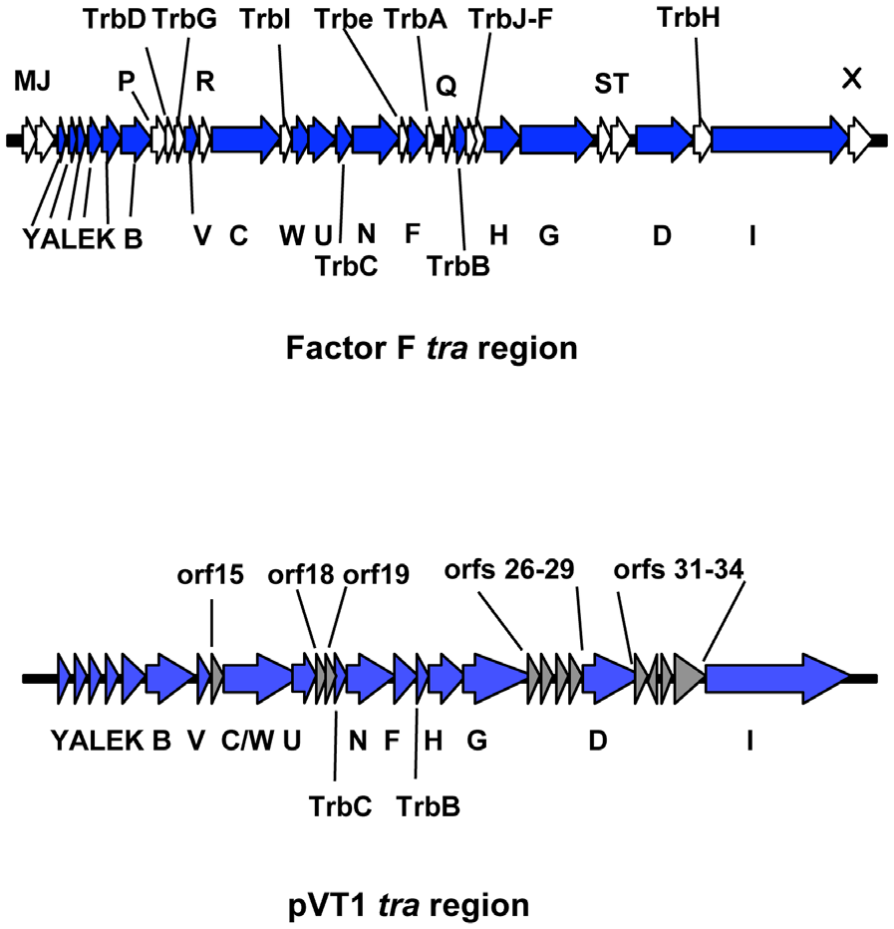
Comparison between the *E. coli* F factor and pVT1 *tra* regions. Genes with function in conjugation and conserved between the two plasmids are indicated by arrows filled in blue. Genes specific to the F plasmid are filled in white. Names of genes are indicated (no prefix: *tra* genes). For the F factor, names of genes which are conserved between the two plasmids are underneath, whereas genes not conserved are above. In the case of pVT1, genes with no predicted functions in conjugation and no corresponding genes in the F factor are filled in grey and are listed above.

Similarity searches on deduced pVT1 ORFs encoded proteins using BlastP showed that, for the vast majority of the ORFs (65/88) significant similarities found were, at first rank, with proteins encoded by plasmids or chromosomal DNA from marine bacteria and mostly from bacteria common in estuarine or coastal environment, such as *Vibrionaceae* and *Shewanellaceae* (Table S1). Together with the variability of the G+C contents across different regions of the plasmid (Fig. 1, inside track), these results suggest that pVT1 evolved through gene transfer between these marine bacteria. Using BlastN, several blocks with similarities to other plasmids from marine bacteria were identified. Significant similarities (between 70 and 90% identities over 30% of the pVT1 genome) were found mainly to the 48.5 kb plasmid pYJ016 from *V. vulnificus* strain YJ016 [44] and the closely related 56.6 kb plasmid pC4602-1 as well as pC4602-2 from *V. vulnificus* strain CECT4602 [21] (data not shown). In addition to this family of plasmids from *V. vulnificus*, the region encompassing ORFs *pVT1_51* to *pVT1_57* (Fig. 2, Table S1), encoding a type I restriction/modification system displayed 67 to 71% identity to plasmid 1 from *Shewanella* sp. ANA-3. Other related plasmids could be identified through the presence of homologs of pVT1-encoded ORFs in various *Vibrionaceae* and *Shewanellacae* plasmids. Genes encoding the conjugative functions of pVT1 have homologs in *Photobacterium profundum* SS9 pPBPR1, also a conjugative plasmid, with the general gene organization being conserved (unpublished results, GenBank accession number CR377818), as well as in pVSAL840, a conjugative plasmid from *Aliivibrio salmonicida* LF118, and several plasmids from *Shewanella baltica* strains. The block encoding pVT1_81 to pVT1_85 is related to pJM1_p25 to pJM1_p28 from *L. anguillarum* plasmid pJM1 [19]. Several other ORFs have similarity with proteins encoded by these marine bacteria plasmids. Figure 2 presents a schematic representation of these homology relationships between pVT1 and a subset of such plasmids. In addition, extensive conservation of synteny between homologous regions of these plasmids (Fig. 3) underscores the close relationships between them. It is also noteworthy that although pVT1 conjugative region is very similar to this of the narrow host range plasmid F (see below and Fig. 4), a similar region is found on several of these plasmids, strongly suggesting that they can be moved from one species to another through conjugation.

Twelve pVT1 ORFs present similarities to transposase genes or fragments of transposases, with six corresponding to potentially functional Insertion Sequences (IS). In several cases, DNA blocks, whose genetic organization was mostly conserved, were flanked by such IS elements or traces of IS (Fig. 2 and 3) suggesting that transposition events played an important role in generating the mosaic structure of pVT1: blocks of DNA can thus be moved around through transposition events involving flanking IS.

In addition, the presence of an RS1 phage element is to be noted. RS1 is a satellite defective phage related to the virulence CTXphi phage of *V. cholerae* [45], [46]. In pVT1, it is inserted in a backbone corresponding to genes from the *V. vulnificus* plasmids pYJ016 and pC4602-1, between *pVT1_88*, an ORF potentially involved in DNA replication/initiation (see below) and the *tra* region.

We also noted the presence of several pseudogenes. For instance, pVT1_81 corresponds to a truncated version of pJM1_p25 of *L. anguillarum*, and the neighboring region between *pVT1_81* and *pVT1_82* contains several short ORFs that encode small peptides with similarities to the rest of pJM1_p25 (one of this fCDS is listed in Fig. 2). pVT1_75 and pVT1_76 correspond to the N-terminal part and the C-terminal part, respectively, of an IS5 family transposase (Table S1).

### pVT1 is a conjugative plasmid related to *V. vulnificus* plasmids pYJ016 and pC4602-1

A 35 kb region very similar to the transfer region of two conjugative plasmids isolated from two different strains of *V. vulnificus*, pYJ016 and pC4602-1 [21] encodes 18 genes sharing similarity with proteins involved in DNA transfer of the *E. coli* F factor (Fig. 1 and 4), the genetic organization of this region being mostly conserved between these two plasmids (Fig. 2).

Conjugation systems, which can transfer DNA between a donor cell and a recipient cell are related to T4SS [47], which, in pathogenic bacteria, can inject effector proteins directly into host cells. Conjugative T4SS capable of nucleic acid transport encode a coupling protein – in our case, TraD - that interacts with the DNA-protein complex (the relaxosome) to guide it to the T4SS at the membrane. The presence of genes encoding a TraD homolog (pVT1_30) and TraI (pVT1_35), a protein required for nicking and unwinding the substrate DNA, strongly suggests that this region encodes a plasmid conjugation system. Compared to the *Escherichia coli* F plasmid *tra* region, the transfer region is lacking genes *traM*, *J*, *P*, *R*, *Q*, *S*, *T* and *X*, as in the case of pYJ016 and pC4602-1 (Fig. 4 and data not shown). However, in addition to *traD* and *I*, all essential genes for pilus synthesis and assembly (*traA*, *L*, *E*, *K*, *B*, *V*, *C*, *W*, *U*, *F* and *G*, and *trbC*), and genes for mating pair stabilization (*traN-G*) are present on pVT1 as they are on pC4602-1 (Figs. 1, 2 and 4; [21]; for a review see [48]).

There is very little divergence between the *tra* regions of the F factor and pVT1, the main differences corresponding to insertion of ORFs of unknown function and the absence of the two entry exclusion genes *traS* and *traT*, normally located between *traG* and *traD* (Fig. 4). Entry exclusion is the property by which a cell that harbors a conjugative plasmid becomes bad recipient for this plasmid or a close relative of it. It has been suggested that exclusion is an essential feature of conjugative plasmids and that it is important for their stability [49]. However, no candidates for this function could be identified in pVT1 *tra* region, or in any of the related conjugative plasmids. Especially, genes present between *traG* and *traD* on these plasmids are not homolog to each other (Fig. 2).

Although the analysis of the *tra* region suggests that conjugation is functional, initial attempts to transfer pVT1 through conjugation to *E. coli* after introduction of a suicide plasmid vector carrying a chloramphenicol cassette [41] in the gene encoding an IS4-like transposase (*pVT1_36*) were unsuccessful, even when we used a restriction minus strain such as DHα-T1 (see Material and Methods). However, we were able to obtain CmR exconjugants after mating VT14 with *V. harveyi* LMG7890, a non pathogenic strain with no described plasmid [42], with a frequency of 7,5±4×10^−4^. Identity of the exconjugants as *V. harveyi* as well as the presence of the Cm resistance cassette was confirmed by PCR, using specific primers. However no plasmid could be extracted from the clones and we could not detect any plasmid sequences, using a variety of pVT1 specific primers (data not shown). This result suggests that, although the plasmid is conjugative, it could not establish itself successfully in the recipient strain. It is then likely that the suicide vector could excise from pVT1 and recombine by homologous recombination between the disrupted IS4 it carries and an IS4 present on the *V. harveyi* genome.

### Plasmid maintenance functions and copy number

**Figure 5:**
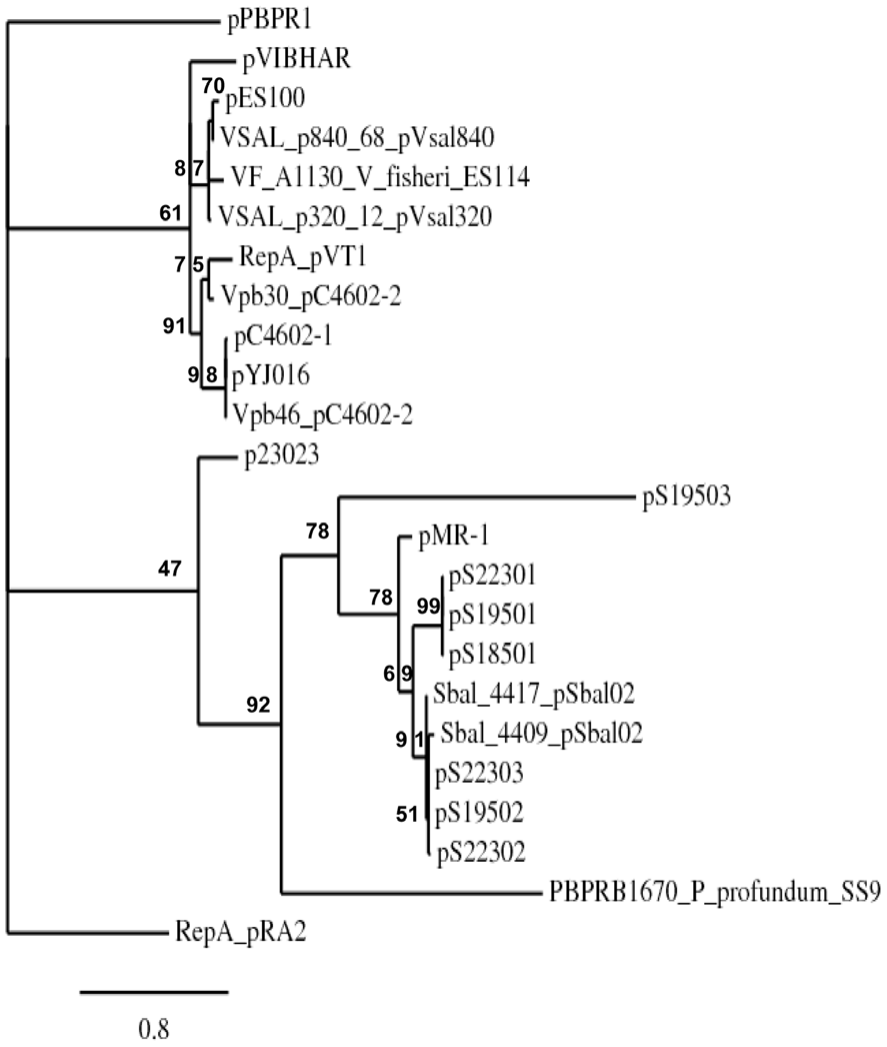
Phylogenic tree of homologous proteins of pVT1_88, including the RepA protein of plasmid pRA2 from *P. alcaligenes,* used as an outgroup. The name of each plasmid carrying an homolog (see also text for details) is indicated. In case of two homologous genes on a plasmid, the full names of the proteins are indicated. In case of chromosome-encoded proteins, the name of the species is indicated in addition to the protein. Plasmid p23023 is from Vibrio sp. BMSA_0011. Significance of each node is assessed by approximate likelihood-ratio tests (aLRT) and expressed as a percentage [62].

Plasmids use the host replication machinery for their own replication but usually encode at least one protein required for initiating replication and capable of recognizing a specific site within the plasmid, used as an origin of replication. The plasmid-encoded protein pVT1_88 showed high similarity to pYJ016-encoded VVP68 and its homologs from pC4602-1, pC4602-2, as well as to conserved proteins from other *Vibrio* plasmids, such as VFB17 from pES100 (*Vibrio fisheri* ES114; accession number YP_207147) and PBPRC0066 from pPBPR1 (*P. profundum*; accession number YP_015520) and also to several proteins encoded by plasmids from *Shewanellaceae* (see Fig. 5 for a phylogenetic analysis). Moreover, these proteins are homologous to the RepA protein encoded by the *Pseudomonas alcaligenes* plasmid pRA2 (Table S1), which was shown to be essential for plasmid replication [50] and appear to define a novel type of replicon. This suggests that pVT1_88 is required for replication of pVT1. However, it is to be noted that, despite the fact that the majority of pVT1_88 homologs are encoded by plasmids, some were encoded on chromosomes (Fig. 5). More surprisingly, since in general a plasmid Rep protein is involved in replicon incompatibility, in strains harboring several plasmids such as *V. vulnificus* CECT4602 (pC4602-1 and pC4602-1), *A. salmonicida* (pVsal840, pVsal320) or *Shewanella baltica* 0S223 (pS22301, pS22302, pS22303), a copy of the gene was found on each of these replicons, as also noted by Hazen *et al*. in the case of *V. vulnificus* CECT4602 [2]. In some cases, a single plasmid could even harbor two copies of the gene (i.e. pC4602-2, pSbal02) (Fig. 5).

Plasmid origins of replication are often linked to genes encoding their initiation protein. However, contrary to the case of pRA2, we were unable to identify, close to this ORF, or elsewhere in the plasmid, iterons or DnaA binding sites, which may have indicated the presence of an origin of replication. An analysis of the G−C/G+C content along the plasmid (the so-called GC skew, [51] failed also to identify a potential origin. When we tried to use a DNA fragment encoding pVT1_88 and encompassing the neighboring regions to confer autonomous replication to a suicide plasmid in a restriction minus strain of *E. coli*, no clones were recovered. Since this could be due to the fact that this replicon is not functional in *E. coli*, as suggested by our inability to introduce pVT1 in *E. coli via* conjugation in the same strain, we tried to transform *V. cholerae* by electroporation with the same construction. No clones were recovered, in contrast to a plasmid carrying a p15A origin, functional in *Vibrionaceae*. Again, this may be due to the requirement for another protein for pVT1 replication and/or to the fact that the plasmid replication origin is located elsewhere.

In addition, we determined the copy number of pVT1: Real Time quantitative PCR was used to determine the ratio of the plasmid encoded genes, *traG* and *traC* to the chromosomal encoded gene *gyrB* [39]. This ratio was of 11.3±2 in case of *traC* and 7,7±1.4 in case of *traG* indicating the copy number to be around 9 (Table 1 and Material and Methods). Although not very high, this pVT1 copy number came as a surprise since large plasmids have generally a very low copy number around one. However, the technique used has been shown to give results comparable to other techniques such as Southern blot [39] and was able to detect a 3 fold decrease of pVT1 copy number following successive passages at sublethal temperature (data not shown). In case of the related *V. vulnificus* plasmids, their copy number was not determined. Future studies aiming at analyzing this novel family of replicons will be required to understand how initiation of replication is controlled.

**Table 1:** Plasmid copy number determination.

A		
Genes	Efficiency	Linearity
*gyrB*	115%	0,999
*traC*	107%	0,999
*traG*	95%	0,998

B						
Intercepts			ΔCt		Copy number	
*gyrB*	*traC*	*traG*	*traC*	*traG*	*traC*	*traG*
16.85	13.44	14.24	3.41	2.61	10.63	6.10
16.81	13.05	13.70	3.76	3.11	13.55	8.63
16.03	12.75	12.97	3.28	3.06	9.71	8.34
**Mean ± SD**:					**11.3±2**	**7.7±1.4**

A: Q-PCR efficiency and linearity for each gene.

B: *traC* and *traG* copy number determination compared to *gyrB*. See Materials and Methods for details of calculation.

Upstream of *pVT1_88*, in the clockwise direction, separated from *pVT1_88/repA* by two ORFs corresponding to truncated transposases, we identified two ORFs, *pVT1_84* and *85* with similarity to *parA* and *parB*. These genes are together important for low copy number plasmid partitioning. ParB binds to a centromer-like site, *parS*, and recruits ParA, which possesses an ATPase activity. The complex is actively involved in partitioning plasmids between the two daughter cells [52].

We also noted the presence of a second *parB* gene on pVT1 (*pVT1_ 43*) not accompanied by a *parA* gene. The same situation occurs in the case of the *Shigella flexneri* virulence plasmid pWR100, where the second *parB*-like gene, *virB*, is required for transcription of a subset of virulence genes [13]. In that case, the authors suggested that this second *parB* gene could have a different function than plasmid partition.

Surprisingly, part of the region encompassing pVT1_81 to _85, and encoding putative partition proteins had also similarities (including up to 91% identities at the nucleotide level) to a chromosomal region of the recently sequenced genome of *Aliivibrio salmonicida* (Hjerde, 2008, unpublished results, accession FM178379). In addition, we found several examples of plasmid sequences present in certain strains of *V. vulnificus*, *Vibrio harveyi* and *P. profondum* on chromosomal regions rather than on plasmids (data not shown), illustrating the exchange of genetic materials between chromosomal and plasmid DNA.

Finally, we identified in pVT1 a putative toxin/antitoxin system. *pVT1_44* encodes a polypeptide with similarity to the *V. cholerae* toxin HigB-2, whereas *pVT1_45* encodes a homolog of HigA-2 (Table S1). *V. cholerae* has two *higBA* loci located on the chromosome II super-integron [53]. HigB toxins inhibit translation by cleaving translated mRNAs [54] and their action is counteracted by the product of a second gene, HigA. Contrary to most known other toxin/antitoxin systems, in *higBA* operons, the toxin is encoded upstream of the antitoxin as it is the case in pVT1. pVT1_45/HigA belongs to the superfamily of HTH-XRE transcriptional regulator and is proposed to be a transcriptional repressor of the HigBA operon [55]. This toxin/antitoxin system is likely to contribute to the stability of pVT1 and its presence may explain our failure to cure *V. tapetis* of its plasmid despite numerous attempts either by acridine orange treatments or by passages at sublethal higher temperature.

### Putative functions encoded by pVT1

No genes with an obvious implication in virulence, such as genes encoding TTSS or T4SS, have been identified on the plasmid. As often the case with plasmid or phage DNA, 38 genes (43% of the total) encode proteins with no known functions, even if a number of them have homologs in other marine bacterial species. However, several of those genes encode potential transcription regulators, envelope proteins and secreted proteins (Table S1) and thus could be important for pathogenesis or adaptation to specific growth conditions.

Appropriate timing of gene expression is important for host-pathogen interactions and acquisition of novel regulators could confer new properties by changing an expression pattern, as in the case of *Vibrio fisheri* [56]. In addition to pVT1_45/HigA-2, pVT1_69 also encodes a putative transcription regulator. pVT1_83 has some similarity to the transcription antitermination factor NusB (as determined by PSI-BLAST, Table S1) and could also regulate gene expression.

Amongst proteins predicted to localize to the envelope, pVT1_46 is an inner membrane protein and belongs to the RDD family of transporter whereas pVT1_77 is a putative outer membrane lipoprotein. pVT1_73 is predicted to have a signal sequence and to be exported to the periplasm by Psort (Material and Methods). It also belongs to the COG0708 family, which corresponds to an exonuclease III. *V. tapetis* has been shown to have a secreted nuclease activity [57]. Whether pVT1_73 is further secreted to the external medium and contribute to such activity remains to be determined.

Finally, pVT1_65 has similarity to the virulence-associated protein MsgA (Table S1), which is a member of the DNA damage inducible protein DinI family. In *E. coli*, DinI contributes to the SOS response by stabilizing RecA filaments [58]. In *Salmonella typhimurium*, MsgA was found to play a role in macrophage survival [59]. Thus, pVT1_65 might be important for virulence in *V. tapetis*, especially as one of the defense mechanism of clams is the killing of the bacteria engulfed in the hemocytes by the production of reactive oxygen species, a response known as an oxidative burst [60].

### Concluding remarks

Altogether, our analysis shows that pVT1 consists of a scaffold related to *V. vulnificus* plasmids such as pC4602-1 or pYJ016, in which several blocks of DNA with homology to regions of *Shewanellaceae* and *Vibrionaceae* plasmids have been inserted, possibly through a series of transposition events. Our results strongly suggest that pVT1 defines, together with several other marine bacteria plasmids, a novel family of replicons, with the relatively high copy number of 9 somewhat surprisingly for conjugative plasmids.

The conjugative region of pVT1 is strikingly similar to that of the narrow-host range plasmid F. However this conjugation system is also found in other *Vibrionaceae* plasmids such as *Vibrio vulnificus* pC4202-1, *Aliivibrio salmonicida* pVSAl840, or *Photobacterium profundum* pPBPR1, as well as in *Shewanellaceae* plasmids such as pS22302 or pS19502 (Fig. 2), implying that these plasmids can promote conjugation amongst such bacteria, hence contributing significantly to genetic exchange.

Another possible source of horizontal gene transfer could be marine phages. One example is the RS1 element found in pVT1 (Fig. 1, Table S1). However, no genes with obvious similarity to phage genes were detected in the plasmid (Table S1). Accordingly it seems that conjugation and recombination was the main source of lateral gene transfer.

Horizontal gene transfer appears to be especially active in *Vibrionaceae* [61] and most probably played an important role in generating the *V. tapetis* conjugative plasmid pVT1. Given the absence of obvious virulence genes in pVT1, the question of how these events may have contributed to pathogenicity emergence and host specificity deserves further investigations, which are currently under way.

#### Nucleotide sequence accession number

The GenBank accession number for the sequence reported in this paper is EU573358.

## Supporting Information

Table S1 Proposed functions of pVT1 ORFs.
(DOC)

## References

[1] Thompson FL, Thompson CC, Vicente AC, Klose KE (2010). Vibrio2009: the third international conference on the biology of Vibrios.. Mol Microbiol.

[2] Hazen TH, Pan L, Gu JD, Sobecky PA (2010). The contribution of mobile genetic elements to the evolution and ecology of Vibrios.. FEMS Microbiol Ecol.

[3] Thompson CC, Vicente AC, Souza RC, Vasconcelos AT, Vesth T (2009). Genomic taxonomy of Vibrios.. BMC Evol Biol.

[4] Paillard C (2004). A short-review of brown ring disease, a vibriosis affecting clams, *Ruditapes philippinarum* and *Ruditapes decussatus*.. Aquatic Living Resources.

[5] Paillard C, Maes P (1990). Étiologie de la maladie de l'anneau brun chez Tapes philippinarum: pathogenicité d'un Vibrio sp.. Comptes Rendus de l'Academie des Sciences, Paris, Serie III.

[6] Borrego JJ, Castro D, Luque A, Paillard C, Maes P (1996). *Vibrio tapetis* sp.nov. the causative agent of the Brown ring disease affecting cultured clams. International.. International Journal of systematic bacteriology.

[7] Jensen S, Samuelsen OB, Andersen K, Torkildsen L, Lambert C (2003). Characterization of strains of *Vibrio splendidus* and *V. tapetis* isolated from corkwing wrasse *Symphodus melops* suffering vibriosis.. Dis Aquat Organ.

[8] Park KI, Paillard C, Le Chevalier P, Choi KS (2006). Report on the occurrence of brown ring disease (BRD) in Manila clam, *Ruditapes philippinarum*, on the west coast of Korea.. Aquaculture.

[9] Reid HI, Duncan HI, Laidler A, Hunter D, Birkbeck TH (2003). Isolation of *Vibrio tapetis* from cultivated Atlantic halibut (*Hipoglossus hipoglossus* L.).. Aquaculture.

[10] Lopez JR, Balboa S, Nunez S, de la Roca E, de la Herran R (2010). Characterization of *Vibrio tapetis* strains isolated from diseased cultured Wedge sole (*Dicologoglossa cuneata* Moreau).. Res Vet Sci.

[11] Paillard C, Korsnes K, Le Chevalier P, Le Boulay C, Harkestad L (2008). *Vibrio tapetis*-like strain isolated from introduced Manila clams *Ruditapes philippinarum* showing symptoms of brown ring disease in Norway.. Dis Aquat Organ.

[12] Heesemann J, Sing A, Trulzsch K (2006). Yersinia's stratagem: targeting innate and adaptive immune defense.. Curr Opin Microbiol.

[13] Buchrieser C, Glaser P, Rusniok C, Nedjari H, D'Hauteville H (2000). The virulence plasmid pWR100 and the repertoire of proteins secreted by the type III secretion apparatus of *Shigella flexneri*.. Mol Microbiol.

[14] Jennison AV, Verma NK (2004). *Shigella flexneri* infection: pathogenesis and vaccine development.. FEMS Microbiol Rev.

[15] Chu C, Chiu CH (2006). Evolution of the virulence plasmids of non-typhoid Salmonella and its association with antimicrobial resistance.. Microbes Infect.

[16] Stork M, Di Lorenzo M, Welch TJ, Crosa LM, Crosa JH (2002). Plasmid-mediated iron uptake and virulence in *Vibrio anguillarum*.. Plasmid.

[17] Walter MA, Potter SA, Crosa JH (1983). Iron uptake system mediated by *Vibrio anguillarum* plasmid pJM1.. J Bacteriol.

[18] Crosa JH (1980). A plasmid associated with virulence in the marine fish pathogen *Vibrio anguillarum* specifies an iron-sequestering system.. Nature.

[19] Di Lorenzo M, Stork M, Tolmasky ME, Actis LA, Farrell D (2003). Complete sequence of virulence plasmid pJM1 from the marine fish pathogen *Vibrio anguillarum* strain 775.. J Bacteriol.

[20] Valiente E, Lee CT, Lamas J, Hor L, Amaro C (2008). Role of the virulence plasmid pR99 and the metalloprotease Vvp in resistance of *Vibrio vulnificus* serovar E to eel innate immunity.. Fish & Shellfish Immunology.

[21] Lee CT, Amaro C, Wu KM, Valiente E, Chang YF (2008). A common virulence plasmid in biotype 2 *Vibrio vulnificus* and its dissemination aided by a conjugal plasmid.. J Bacteriol.

[22] Le Roux F, Labreuche Y, Davis BM, Iqbal N, Mangenot S (2010). Virulence of an emerging pathogenic lineage of *Vibrio nigripulchritudo* is dependent on two plasmids.. Environ Microbiol. 2010 Sep 6. [Epub ahead of print].

[23] Reynaud Y, Saulnier D, Mazel D, Goarant C, Le Roux F (2008). Correlation between detection of a plasmid and high-level virulence of *Vibrio nigripulchritudo*, a pathogen of the shrimp *Litopenaeus stylirostris*.. Appl Environ Microbiol.

[24] Sorum H, Roberts MC, Crosa JH (1992). Identification and cloning of a tetracycline resistance gene from the fish pathogen Vibrio salmonicida.. Antimicrob Agents Chemother.

[25] Zhang R, Gu JD (2009). Complete sequence of plasmid pMP1 from the marine environmental *Vibrio vulnificus* and location of its replication origin.. Mar Biotechnol (NY).

[26] Zhang R, Wang Y, Leung PC, Gu JD (2007). pVC, a small cryptic plasmid from the environmental isolate of *Vibrio cholerae* MP-1.. J Microbiol.

[27] Le Roux F, Davis BM, Waldor MK (2010). Conserved small RNAs govern replication and incompatibility of a diverse new plasmid family from marine bacteria.. Nucleic Acids Res. 2010 Oct 4. [Epub ahead of print].

[28] Pan L, Leung PC, Gu JD (2010). A new ColE1-like plasmid group revealed by comparative analysis of the replication proficient fragments of Vibrionaceae plasmids.. J Microbiol Biotechnol.

[29] Castro D, Romalde JL, Vila J, Magarinos B, Luque A (1997). Intraspecific characterization of *Vibrio tapetis* strains by use of pulsed-field gel electrophoresis, ribotyping, and plasmid profiling.. Appl Environ Microbiol.

[30] Le Chevalier P, Le Boulay C, Paillard C (2003). Characterization by restriction fragment length polymorphism and plasmid profiling of *Vibrio tapetis* strains.. J Basic Microbiol.

[31] Sambrook J, Russell D (2001). Molecular Cloning - A laboratory manual..

[32] Leblond P, Fischer G, Francou FX, Berger F, Guerineau M (1996). The unstable region of *Streptomyces ambofaciens* includes 210 kb terminal inverted repeats flanking the extremities of the linear chromosomal DNA.. Mol Microbiol.

[33] Shizuya H, Birren B, Kim UJ, Mancino V, Slepak T (1992). Cloning and stable maintenance of 300-kilobase-pair fragments of human DNA in *Escherichia coli* using an F-factor-based vector.. Proc Natl Acad Sci U S A.

[34] Benes V, Hostomsky Z, Arnold L, Paces V (1993). M13 and pUC vectors with new unique restriction sites for cloning.. Gene.

[35] Besemer J, Borodovsky M (2005). GeneMark: web software for gene finding in prokaryotes, eukaryotes and viruses.. Nucleic Acids Res.

[36] Juncker AS, Willenbrock H, Von Heijne G, Brunak S, Nielsen H (2003). Prediction of lipoprotein signal peptides in Gram-negative bacteria.. Protein Sci.

[37] Vallenet D, Engelen S, Mornico D, Cruveiller S, Fleury L (2009). MicroScope: a platform for microbial genome annotation and comparative genomics.. Database (Oxford).

[38] Dereeper A, Guignon V, Blanc G AS, Buffet S, Chevenet F, Dufayard JF, Guindon S, Lefort V, Lescot M, Claverie JM, Gascuel O (2008). Phylogeny.fr: robust phylogenetic analysis for the non-specialist.. Nucleic Acids Res.

[39] Providenti MA, O'Brien JM, Ewing RJ, Paterson ES, Smith ML (2006). The copy-number of plasmids and other genetic elements can be determined by SYBR-Green-based quantitative real-time PCR.. J Microbiol Methods.

[40] Demarre G, Guerout AM, Matsumoto-Mashimo C, Rowe-Magnus DA, Marliere P (2005). A new family of mobilizable suicide plasmids based on broad host range R388 plasmid (IncW) and RP4 plasmid (IncPalpha) conjugative machineries and their cognate *Escherichia coli* host strains.. Res Microbiol.

[41] Lakhal F, Bury-Mone S, Nomane Y, Le Goic N, Paillard C (2008). DjlA, a membrane-anchored DnaJ-like protein, is required for cytotoxicity of clam pathogen *Vibrio tapetis* to hemocytes.. Appl Environ Microbiol.

[42] Travers MA, Le Bouffant R, Friedman CS, Buzin F, Cougard B (2009). Pathogenic *Vibrio harveyi*, in contrast to non-pathogenic strains, intervenes with the p38 MAPK pathway to avoid an abalone haemocyte immune response.. J Cell Biochem.

[43] Biskri L, Bouvier M, Guerout AM, Boisnard S, Mazel D (2005). Comparative study of class 1 integron and *Vibrio cholerae* superintegron integrase activities.. J Bacteriol.

[44] Chen CY, Wu KM, Chang YC, Chang CH, Tsai HC (2003). Comparative genome analysis of *Vibrio vulnificus*, a marine pathogen.. Genome Res.

[45] Faruque SM, Asadulghani, Kamruzzaman M, Nandi RK, Ghosh AN (2002). RS1 element of Vibrio cholerae can propagate horizontally as a filamentous phage exploiting the morphogenesis genes of CTXphi.. Infect Immun.

[46] Davis BM, Kimsey HH, Kane AV, Waldor MK (2002). A satellite phage-encoded antirepressor induces repressor aggregation and cholera toxin gene transfer.. Embo J.

[47] Lawley TD, Klimke WA, Gubbins MJ, Frost LS (2003). F factor conjugation is a true type IV secretion system.. FEMS Microbiol Lett.

[48] Frost LS, Ippen-Ihler K, Skurray RA (1994). Analysis of the sequence and gene products of the transfer region of the F sex factor.. Microbiol Rev.

[49] Garcillan-Barcia MP, de la Cruz F (2008). Why is entry exclusion an essential feature of conjugative plasmids?. Plasmid.

[50] Kwong SM, Yeo CC, Chuah D, Poh CL (1998). Sequence analysis of plasmid pRA2 from *Pseudomonas alcaligenes* NCIB 9867 (P25X) reveals a novel replication region.. FEMS Microbiol Lett.

[51] Grigoriev A (1998). Analyzing genomes with cumulative skew diagrams.. Nucleic Acids Res.

[52] Leonard TA, Moller-Jensen J, Lowe J (2005). Towards understanding the molecular basis of bacterial DNA segregation.. Philos Trans R Soc Lond B Biol Sci.

[53] Budde PP, Davis BM, Yuan J, Waldor MK (2007). Characterization of a *higBA* toxin-antitoxin locus in *Vibrio cholerae*.. J Bacteriol.

[54] Christensen-Dalsgaard M, Gerdes K (2006). Two *higBA* loci in the *Vibrio cholerae* superintegron encode mRNA cleaving enzymes and can stabilize plasmids.. Mol Microbiol.

[55] Gerdes K, Christensen SK, Lobner-Olesen A (2005). Prokaryotic toxin-antitoxin stress response loci.. Nat Rev Microbiol.

[56] Mandel MJ, Wollenberg MS, Stabb EV, Visick KL, Ruby EG (2009). A single regulatory gene is sufficient to alter bacterial host range.. Nature.

[57] Borrego JJ, Luque A, Castro D, Santamaria JA, Martinez-Manzares E (1996). Virulence factors of the Vibrio P1, the causative agent of brown ring disease in the manila clam, *Ruditapes philippinarum*.. Aquat Living Resour.

[58] Lusetti SL, Voloshin ON, Inman RB, Camerini-Otero RD, Cox MM (2004). The DinI protein stabilizes RecA protein filaments.. J Biol Chem.

[59] Gunn JS, Alpuche-Aranda CM, Loomis WP, Belden WJ, Miller SI (1995). Characterization of the *Salmonella typhimurium pagC/pagD* chromosomal region.. J Bacteriol.

[60] Bugge DM, Hegaret H, Wikfors GH, Allam B (2007). Oxidative burst in hard clam (*Mercenaria mercenaria*) haemocytes.. Fish Shellfish Immunol.

[61] Dryselius R, Kurokawa K, Iida T (2007). Vibrionaceae, a versatile bacterial family with evolutionarily conserved variability.. Res Microbiol.

[62] Anisimova M, Gascuel O (2006). Approximate likelihood-ratio test for branches: A fast, accurate, and powerful alternative.. Syst Biol.

