# Supplementary material for: Evidence for the Role of Horizontal Transfer in Generating pVT1, a Large Mosaic Conjugative Plasmid from the Clam Pathogen, Vibrio tapetis
Source: PLoS One. 2011 Feb 4;6(2):e16759. doi: 10.1371/journal.pone.0016759 (PMC3033894; doi:10.1371/journal.pone.0016759)
Supplement: Table S1 — Proposed functions of pVT1 ORFs. (DOC) [file pone.0016759.s001.doc]

**SUPPORTING INFORMATION**

**Table S1**

| ORF Name/gene | Coordinates  (start to stop codon) | Length (amino  acids) | Proposed function | Evidences:  Homolog(s)^1^; alignment length/length of subject(% of positive); E value; conserved domain, signature, other |
| --- | --- | --- | --- | --- |
| pVT1_1 | 267-494 | 75 | Hypothetical protein |  |
| pVT1_2 | 764-1051 | 95 | Hypothetical protein |  |
| pVT1_3/*rstR* | c1200-1598*^2^ | 132 | RS2 element transcriptional regulator RstR | RstR-like protein [*Vibrio* phage CTX]; 94/132(56%); 4e-07; cd00093, Helix-turn-helix XRE-family like proteins |
| pVT1_4/*rstA1* | 1740-2858 | 372 | Phage replication protein RstA1 | RstA1 [*Vibrio cholerae* 623-39]; 372/372(84%); 3e-160; TIGR01629, phage/plasmid replication protein, gene II/X family |
| pVT1_5/*rstB1* | 2871-3152 | 93 | Phage DNA binding protein RstB1 | RstB1 [*Vibrio cholerae* 623-39]; 94/94(80%); 1e-28; pfam02303, Phage_DNA_bind, Helix-destabilizing protein |
| pVT1_6 | 3149-3502 | 117 | Conserved protein of unknown function | Predicted protein [*Vibrio cholerae* RC385]; 111/115(60%); 4e-17 |
| pVT1_7/*rstC* | 3572-3793 | 73 | RstC protein | RstC protein [*Vibrio cholerae* V51];  65/74(64%); 2e-04 |
| pVT1_8/*traY* | 4062-4241 | 59 | Conjugative protein TraY | Hypothetical protein pC46021_46 [*Vibrio vulnificus*, pC4602-1]; 60/62(85%); 7e-17; pfam05509, TraY family |
| pVT1_9/*traA* | 4805-5092 | 95 | Conjugative protein TraA | Putative conjugative transfer protein TraA [*Vibrio vulnificus*, pC4602-1];  95/95(86%); 9e-36; TIGR02758, TraA, type IV conjugative transfer system pilin TraA |
| pVT1_10/*traL* | 5102-5404 | 100 | Conjugative protein TraL | Putative conjugative transfer protein TraL [*Vibrio vulnificus* YJ016, pYJ016]; 100/100(84%); 4e-39; pfam07178, TraL protein |
| pVT1_11/*traE* | 5414-5989 | 191 | Conjugative pilus assembly protein TraE | Putative conjugative transfer protein TraE [Vibrionales bacterium  SWAT-3]; 190/191(77%); 2e-64; PRK13726, conjugal transfer pilus assembly protein TraE |
| pVT1_12/*traK* | 6075-6767 | 230 | Conjugative protein TraK | Putative conjugative transfer protein TraK [*Vibrio vulnificus* YJ016, pYJ016]; 230/261(85%); PRK13736, conjugal transfer protein TraK |
| pVT1_13/*traB* | 6757-8265 | 502 | Conjugative pilus assembly protein TraB | Putative conjugative transfer protein TraB [*Vibrio vulnificus* pC4602-1];  510/510(76%); 0.0; PRK13729, conjugal transfer pilus assembly protein TraB |
| pVT1_14/*traV* | 8269-8649 | 126 | Conjugative lipoprotein TraV | Hypothetical protein pC46021_49 [*Vibrio vulnificus*, pC4602-1]; 122/130(73%); 2e-35; pfam09676, TraV, Type IV conjugative transfer system lipoprotein, outer membrane protein (lipoP) |
| pVT1_15 | 8663-9025 | 120 | Hypothetical protein |  |
| pVT1_16/*traCW* | 9041-12286 | 1081 | Fused protein TraC-TraW | Putative conjugative transfer protein TraC [*Vibrio vulnificus* YJ016, pYJ016]- putative conjugative transfer protein TraW [*Vibrio vulnificus* YJ016, pYJ016]^3^; 796/850(88%)-270/265(78%);  0.0-4e-96; PRK13721, conjugal transfer ATP-binding protein TraC-PRK13738  conjugal transfer pilus assembly protein TraW |
| pVT1_17/*traU* | 12412-13422 | 336 | Conjugative protein TraU | Putative conjugative transfer protein TraU [Vibrionales bacterium  SWAT-3]; 313/330(93%); 2e-180; PRK13737,  conjugal transfer pilus assembly protein TraU |
| pVT1_18 | 13496-13753 | 85 | Conserved protein of unknown function | Hypothetical protein P99018ORF_172 [*Photobacterium damselae* subsp. *piscicida*]; 45/87(73%); 6e-09 |
| pVT1_19 | 13750-13974 | 74 | Conserved protein of unknown function | Hypothetical protein VVP13 [*Vibrio vulnificus* YJ016, pYJ016]; 70/77(67%); 6e-13 |
| pVT1_20/*trbC* | 13993-14700 | 235 | Conjugative pilus assembly protein TrbC | Putative conjugative transfer protein TrbC [*Vibrio vulnificus*, pC4602-1]; 220/237(69%); 3e-61; PRK13730, conjugal transfer pilus assembly protein TrbC |
| pVT1_21/*traN* | 14714-16444 | 576 | Mating pair stabilization protein TraN | Putative conjugative transfer protein TraN [*Vibrio vulnificus* YJ016, pYJ016]; 575/577(74%); 0.0; PRK12355, mating pair stabilization protein |
| pVT1_22/*traF* | 16492-17277 | 262 | Conjugative protein TraF | Putative conjugative transfer protein TraF [*Vibrio vulnificus* YJ016, pYJ016]; 266/288(86%); 3e-116; PRK13703, conjugal pilus assembly protein TraF |
| pVT1_23/*trbB* | 17367-17780 | 137 | Conjugative pilus assembly protein TrbB, thiol-disulfide isomerase | Putative conjugative transfer protein TrbB [Vibrionales bacterium  SWAT-3]; 137/137(77%); 1e-45; PRK13728, conjugal transfer protein TrbB |
| pVT1_24/*traH* | 17777-19108 | 443 | Conjugative pilus assembly protein TraH | Putative conjugative transfer protein TraH [*Vibrio vulnificus* YJ016, pYJ016]; 443/468(84%); 0.0; PRK13723, conjugal transfer pilus assembly protein TraH |
| pVT1_25/*traG* | 19186-22017 | 943 | Mating pair stabilization protein TraG | Putative conjugative transfer protein TraG [*Vibrio vulnificus* YJ016, pYJ016]; 941/936(83%); 0.0; PRK13735, conjugal transfer mating pair stabilization protein TraG |
| pVT1_26 | 22110-22640 | 176 | Conserved protein of unknown function | Hypothetical protein VVP21 [*Vibrio vulnificus* YJ016, pYJ016]; 177/177(83%); 1e-74 |
| pVT1_27 | 22665-22892 | 75 | Conserved protein of unknown function | Hypothetical protein pC46021_57 [*Vibrio vulnificus* pC4602-1]; 70/81(81%); 5e-19 |
| pVT1_28 | 23449-24034 | 195 | Hypothetical protein |  |
| pVT1_29 | 24060 24452 | 130 | Hypothetical protein |  |
| pVT1_30/*traD* | 24563-26668 | 701 | Conjugative coupling factor TraD | Putative conjugative transfer protein TraD [*Vibrio vulnificus* YJ016, pYJ016]; 695/699(94%); 0.0; PRK13700, conjugal transfer protein TraD |
| pVT1_31 | 26736-26915 | 59 | Hypothetical protein |  |
| pVT1_32 | c26927-27131 | 68 | Hypothetical protein |  |
| pVT1_33 | 27132-27371 | 79 | Hypothetical protein |  |
| pVT1_34 | 27560-29083 | 507 | Hypothetical protein |  |
| pVT1_35/*traI* | 29174-34951 | 1925 | Relaxase TraI | Putative conjugative transfer protein TraI [*Vibrio vulnificus*, pC4602-1]; 1925/1924(91%); 0.0; PRK13709, conjugal transfer nickase/helicase TraI |
| pVT1_36 | 35052-36374 | 440 | IS4 family transposase | Hypothetical protein VIBHAR_01079 [*Vibrio harveyi* ATCC BAA-1116]; 414/440(67%); 3e-105; pfam01609, Transposase_11, Transposase DDE domain, IS4 family^4^ |
| pVT1_37 | c36398-36757 | 119 | Conserved protein of unknown function | Hypothetical protein VVP27 [*Vibrio vulnificus* YJ016, pYJ016]; 119/119(94%); 3e-53 |
| pVT1_38 | 37077-37301 | 74 | Hypothetical protein |  |
| pVT1_39/*pilT* | 37349-37792 | 147 | Lytic transglycosylase PilT | Putative PilT protein [*Vibrio vulnificus*, pR99]; 148/148(85%);  1e­­-58; PRK13722, lytic transglycosylase |
| pVT1_40 | c37854-38189 | 111 | Conserved protein of unknown function | Hypothetical protein VSWAT3_22035 [Vibrionales bacterium SWAT-3]; 102/111(60%); 4e-33 |
| pVT1_41 | c38274-38660 | 128 | Conserved protein of unknown function | Hypothetical protein VSWAT3_25719 [Vibrionales bacterium SWAT-3]; 127/128(82%); 1e-44 |
| pVT1_42 | c38684-39028 | 114 | Conserved protein of unknown function | Hypothetical protein VSWAT3_25724 [Vibrionales bacterium SWAT-3]; 114/118(67%); 6e-25 |
| pVT1_43/*parB2* | c39712-41568* | 618 | Partition protein ParB like | ParB domain protein nuclease [*Shewanella baltica* OS223, pS22303];  599/624(66%); 1e-145; PRK13832, plasmid partitioning protein |
| pVT1_44/*higB* | 42416*-42748 | 110 | Addiction module toxin HigB, RelE related | Toxin HigB-2 [*Vibrio cholerae* CIRS 101]; 79/79(93%); 5e-34 |
| pVT1_45/*higA* | 42735*-43049 | 104 | Addiction module antitoxin HigA, Putative transcriptional repressor | Virulence gene repressor RsaL [*Vibrio fischeri* ES114]; 104/104(95%); 8e-49; similarity to protein VCA0469-HigA-2 [Vibrio cholerae O1 biovar eltor str. N16961]; 104/104(93%); 3e-46; COG2944, Predicted transcriptional regulator, |
| pVT1_46 | 43465-43956 | 163 | Conserved membrane protein of unknown function; putative transporter | Predicted membrane protein/domain [*Vibrio alginolyticus* 12G01]; 163/163(98%); 3e-91; pfam06271, RDD family |
| pVT1_47/*istA* | 44549-46084 | 511 | IS21 family transposase subunit IstA | Transposase [Vibrionales bacterium SWAT-3]; 510/510(88 %); 0.0;  COG4584, Transposase and inactivated derivatives, IS21 family |
| pVT1_48/*istB* | 46094-46834 | 246 | IS21 family transposase subunit IstB | Putative transposase subunit [Vibrionales bacterium SWAT-3]; 246/246(93%); 4e-115; pfam01695, IstB, IstB-like ATP binding protein, IS21 family |
| pVT1_49 | 46979-47446 | 155 | Conserved protein of unknown function | Hypothetical protein Rleg_1841 [*Rhizobium leguminosarum* bv. *trifolii* WSM1325]; 104/154(55%); 3e-08 |
| pVT1_50 | 47524*-48066 | 180 | Hypothetical protein |  |
| pVT1_51 | 48330-48923 | 197 | IS1004 family transposase | Phage integrase family protein [Vibrionales bacterium SWAT-3];  197/197(96%); 2e-101; cd01182, DNA breaking-rejoining enzymes, integrase/recombinases, C-terminal catalytic domain, IS1004 family |
| pVT1_52 | 49111-49515 | 134 | S-adenosylhomocysteine hydrolase | S-adenosylhomocysteine hydrolase [*Vibrio vulnificus* CMCP6]; 134/150(95%); 9e-65 |
| pVT1_53 | 49518-50459 | 313 | Conserved protein of unknown function | Hypothetical protein VMA_002597 [*Vibrio mimicus* VM223]; 313/313(88%); 2e-137; pfam08843, DUF1814, Protein of unknown function |
| pVT1_54/*hsdM* | 50467-53061 | 864 | Type I restriction-modification system N6-Methylase | Type I restriction-modification system M subunit [*Vibrio mimicus* VM223]; 855/860(89%); 0.0;  COG0286, HsdM, Type I restriction-modification system methyltransferase subunit |
| pVT1_55/*hsdS* | 53054-54313 | 419 | Type I restriction modification system DNA specificity subunit HsdS | Restriction modification system DNA specificity subunit [Yersinia pseudotuberculosis YPIII] ; 416/410(60%); 1e-79; PRK09737, EcoKI restriction-modification system protein HsdS |
| pVT1_56/*hsdR* | 54475*-57591 | 1038 | Type I restriction- modification system deoxyribonuclease  subunit HsdR | Type I site-specific deoxyribonuclease HsdR family [*Vibrio mimicus* VM223]; 1035/1038(89%); 0.0;  COG0610, Type I site-specific restriction-modification system, R (restriction) subunit and related helicases |
| pVT1_57 | c58202-59176 | 324 | Putative bacteriophage abortive infection protein | Conserved hypothetical protein [*Acinetobacter* sp. RUH2624]; 316/314(70%); 7e-93; pfam07751, Abi_2 family involved in bacteriophage resistance |
| pVT1_58 | 59857-60072 | 71 | Hypothetical protein |  |
| pVT1_59 | 60128-62151 | 677 | Hypothetical protein |  |
| pVT1_60 | c62574-62870 | 98 | Hypothetical protein |  |
| pVT1_61 | c62920-63099 | 59 | Hypothetical protein |  |
| pVT1_62 | c63134-63598 | 154 | Conserved protein of unknown function | VSWAT3_25934 [Vibrionales bacterium SWAT-3]; 154/169(54%); 3e-22 |
| pVT1_63 | c63614-64084 | 156 | Hypothetical protein | Hypothetical protein pJM1_p22 [*Listonella anguillarum*](N-terminal part)-YbaB [*Photorhabdus luminescens*](C-terminal part); 35/128(77%)-43/136(55%); 1e-04 -4e-04 |
| pVT1_64 | 64452-64613 | 53 | Hypothetical protein |  |
| pVT1_65/*msgA* | c64610-64897 | 95 | DinI-like protein MsgA | Putative DNA-damage-inducible protein [*Aliivibrio salmonicida* LFI1238 pVSAL320]; 76/76(77%;) 6e-17 ; similarity to Virulence associated protein MsgA [Erwinia billingiae Eb661]; 63/81 (63%); 5e-06;  PRK10597, DNA damage-inducible protein I |
| pVT1_66 | c64986-65195 | 69 | Conserved protein of unknown function | Hypothetical protein BMSA_0026 [*Vibrio* sp. 23023]; 47/86(76%); 2e-08 |
| pVT1_67 | 65535-66137 | 200 | Conserved protein of unknown function | Hypothetical protein VIBHAR_02592 [*Vibrio harveyi* ATCC BAA-1116];  192/193(86%); 3e-75; pfam11140, DUF2913, Protein of unknown function |
| pVT1_68 | c66269-66850* | 193 | IS6 family transposase (C-terminal part) | Putative transposase PBPRB1653 [*Photobacterium profundum* SS9]; 155/161(96%); 1e-77; COG3316, Transposase and inactivated derivatives, IS6 family |
| pVT1_69 | c67198-67614 | 138 | Putative transcriptional regulator | Hypothetical protein PBPRB1763 [*Photobacterium profundum* SS9]; 138/141(78%); 2e-43; COG5499, transcription regulator containing HTH domain |
| pVT1_70 | c67623-67952 | 109 | Conserved protein of unknown function | Hypothetical protein PBPRB1762 [*Photobacterium profundum* SS9]; 104/105(78%); 6e-32; COG4680, Uncharacterized protein conserved in bacteria |
| pVT1_71/*tnpR* | 68086-68748 | 220 | Putative resolvase of Tn3 transposon family | Resolvase domain-containing protein [*Shewanella baltica* OS155]; 203/212(87%); 3e-84; COG1961, PinR, Site-specific recombinases, DNA invertase Pin homologs, Tn3 family resolvase TnpR |
| pVT1_72 | c68866-69102 | 78 | Hypothetical protein |  |
| pVT1_73 | 69525-70388 | 287 | Putative exported nuclease | Endonuclease/exonuclease/phosphatase [*Serratia proteamaculans* 568];  256/298(59%); 6e-42; COG0708, Exonuclease III |
| pVT1_74 | 70575-71312 | 245 | IS6 family transposase | Putative transposase [*Photobacterium profundum* SS9, pPBPR1]; 245/245(97%); 3e-135; COG3316, Transposase and inactivated derivatives, IS6 family |
| pVT1_75 | 71589-72068 | 159 | IS5 family transposase (N-terminal fragment) | Hypothetical transposase [*Photobacterium profundum* SS9]; 150/265(91%); 1e-71, IS5 family |
| pVT1_76 | 72149-72565 | 138 | IS5 family transposase (C-terminal fragment) | Putative transposase [*Photobacterium profundum SS9*, pPBPR1]; 138/214(94%);  8e-69; pfam01609, Transposase_11, Transposase DDE domain, IS5 family (truncated) |
| pVT1_77 | 73090-73524 | 144 | Putative outer membrane lipoprotein | Hypothetical phage protein [*Yersinia enterocolitica* subsp. enterocolitica 8081]; 139/140(49%); 9e-12; lipoP prediction: outer membrane lipoprotein |
| pVT1_78 | c73680-74417 | 245 | IS6 family transposase | Putative transposase [*Photobacterium profundum* SS9, pPBPR1]; 245/245(97%); 7e-136; COG3316, Transposase and inactivated derivatives, IS6 family |
| pVT1_79 | c74489-74770 | 93 | Conserved protein of unknown function | Hypothetical protein Shew185_1874 [*Shewanella baltica* OS185]; 82/86(56%); 5e-08 |
| pVT1_80 | c74780-75391 | 203 | Conserved protein of unknown function (C-terminal fragment) | Hypothetical protein Shew185_1873 [*Shewanella baltica* OS185]; 204/290(65%); 2e-43 |
| pVT1_81 | c75406-75624 | 72 | Conserved protein of unknown function (N-terminal fragment) | Hypothetical protein Shew185_1873 [*Shewanella baltica* OS185]; 47/290(72%); 4e-05 |
| pVT1_82 | c76285-76656 | 123 | Conserved protein of unknown function | Hypothetical protein VSAL_I0061 [*Aliivibrio salmonicida* LFI1238]; 123/123(98%); 5e-62 |
| pVT1_83 | c77104-77508 | 134 | Putative transcription antitermination factor | Hypothetical protein VSAL_I0060 [*Aliivibrio salmonicida* LFI1238]; 134/134(94%); 4e-64 ; similarity to NusB antitermination factor [*Roseiflexus castenholzii* DSM 13941] by PSI-Blast : 54/134(40%) ; 7e-27 |
| pVT1_84/*parB1* | c77498-78472 | 324 | Partition protein ParB like | Putative chromosome partitioning protein ParB VSAL_I0059 [*Vibrio salmonicida* LFI1238]; 324/324(92%); 6e-164; TIGR00180, ParB-like partition proteins |
| pVT1_85/*parA* | c78469-79266 | 265 | Partition protein ParA | Putative ParA chromosome partitioning protein VSAL_I0058 [*Aliivibrio salmonicida*  LFI1238]; 265/265(96%); 6e-144; COG1192, Soj, ATPases involved in chromosome partitioning |
| pVT1_86 | c79311-79598* | 95 | Fragment of Tn3 family transposase | Conserved hypothetical protein [*Legionella drancourtii* LLAP12];  38/1029(73%) ; 4e-11 ; similarity to Tn3 transposase of ISShfr9 from *Shewanella frigidimarina*: 61/992(59%) ; 1e-04 (IS finder) |
| pVT1_87 | 79533-79760 | 76 | Fragment of IS630 family transposase | Resolvase, N-terminal domain [*Shewanella* sp. W3-18-1]; 36/206(83%); 2e-05; pfam01710, Transposase_14 |
| pVT1_88/*repA* | c80849-81733 | 294 | Putative replication protein | Hypothetical protein VCJ_000584 [*Vibrio* sp. RC341]; 276/276(79%);7e-107; similarity by PSI-Blast to RepA [*Vibrio ordalii*, plasmid pMJ101]; 296/293(31%); 3e-47 and to RepA [*Pseudomonas alcaligenes*, plasmid pRA2]; 180/276(38%) ; 8e-14 |

1) The indicated similarity is to the closest homolog in the database, except in case of a more informatively annotated homolog. BlastP was run on the NCBI nr protein database, with no conditional adjustments. Other analyses were as indicated in Material and Methods

2)* indicates that the ORF starts with a GTG or a CTG codon

3) This ORF encodes a fusion of TraC (N-terminal part) and TraW (C-terminal part); similarities to both parts are indicated

4) Putative transposases where further analyzed by Blast on the IS finder database, allowing family identification
